# Validation of Liquid Chromatography Coupled with Tandem Mass Spectrometry for the Determination of 12 Tyrosine Kinase Inhibitors (TKIs) and Their Application to Therapeutic Drug Monitoring in Adult and Pediatric Populations

**DOI:** 10.3390/pharmaceutics16010005

**Published:** 2023-12-19

**Authors:** Marie Bellouard, Jean Donadieu, Pauline Thiebot, Etienne Giroux Leprieur, Philippe Saiag, Isabelle Etting, Pamela Dugues, Emuri Abe, Jean-Claude Alvarez, Islam-Amine Larabi

**Affiliations:** 1 Toxicology Laboratory, Raymond Poincaré Hospital, AP-HP, 92380 Garches, France; isabelle.etting@aphp.fr (I.E.); pamela.dugues@aphp.fr (P.D.); emuri.abe@aphp.fr (E.A.); islamamine.larabi@aphp.fr (I.-A.L.); jean-claude.alvarez@aphp.fr (J.-C.A.); 2 Pediatric Hemato-Oncology Department, Trousseau Hospital, AP-HP, 75012 Paris, France; jean.donadieu@aphp.fr; 3 Toxicology Laboratory, Lariboisière Hospital, AP-HP, 75010 Paris, France; pauline.thiebot@aphp.fr; 4 Pneumology Department, Ambroise Paré Hospital, AP-HP, 92100 Boulogne-Billancourt, France; etienne.giroux-leprieur@aphp.fr; 5 Dermatology Department, Ambroise Paré Hospital, AP-HP, 92100 Boulogne-Billancourt, France; philippe.saiag@aphp.fr; 6 Inserm U-1018, CESP, Team MOODS, Plateform MasSpecLab, Paris-Saclay/Versailles University, 78180 Montigny-le-Bretonneux, France

**Keywords:** tyrosine kinase inhibitors, LC-MS/MS, histiocytosis, melanoma, non-small cell lung cancer, therapeutic drug monitoring

## Abstract

Tyrosine kinase inhibitors (TKIs) are used as targeted cancer therapies in adults and have an off-label pediatric application for the treatment of Langerhans cell histiocytosis. A multitarget LC-MS/MS method was developed and validated for the determination of alectinib, alectinib-M4, binimetinib, cobimetinib, crizotinib, dabrafenib, encorafenib, imatinib, lorlatinib, osimertinib, AZ5104, and trametinib. A total of 150 µL of internal standard methanolic solution was added to 50 µL of plasma sample to precipitate proteins. After centrifugation, 10 µL of the supernatant was injected into the chromatographic system. The chromatographic separation was conducted on a Kinetex C18 Polar column with a gradient of 2 mM ammonium formate in 0.1% formic acid and acetonitrile over 5 min. Limits of detection and quantification, linearity, accuracy, precision, selectivity, carryover, matrix effect, recovery, and stability were evaluated and satisfied EMA guidelines on bioanalytical methods. This method has been successfully applied to the therapeutic drug monitoring (TDM) of adults with melanoma and lung cancer, as well as children with histiocytosis, to improve the pharmacokinetic data for these drugs, with the aim of enhancing the therapeutic management and follow-up of patients. Blood concentrations of trametinib and binimetinib were different in the two groups, highlighting the age-related inter-individual variability of these molecules and the need for TDM.

## 1. Introduction

Tyrosine kinase inhibitors (TKIs) are targeted therapies of choice in the treatment of some cancers by inhibiting tyrosine kinase and, thus, a signaling pathway involved in the mechanism of tumor proliferation [1]. These small peptide compounds possess selective inhibition against various kinases, including BRAF, MEK, EGFR, VEGFR, BCR-ABL, PDGFR, and ALK. Their utility extends to diverse areas such as melanoma, non-small cell lung cancer (NSCLC), colorectal cancer, and leukemia [2]. Off-label, some of them (vemurafenib, dabrafenib, trametinib, and cobimetinib) can be used in Langerhans cell histiocytosis (LCH) in children with the BRAF^V600E^ mutation [3,4,5], a rare disease characterized by abnormal macrophage proliferation and granulomatous lesions composed of CD1a^+^CD207^+^ histiocytes [6]. 

Due to the inter-individual variability in drug responses, and in order to verify compliance and improve pharmacokinetic knowledge of these molecules, there is a growing interest in implementing therapeutic drug monitoring (TDM) for these drugs [7,8,9]. In addition, their use is restricted due to tolerance issues such as skin, digestive, and cardiovascular disorders [10,11]. This requires close monitoring. Finally, knowledge of the target concentrations of TKIs is limited and shows heterogeneous minimum effective concentrations, ranging from 10 ng/mL (trametinib) to 1000 ng/mL (imatinib) [12], depending on the molecule.

Thus, the development of sensitive, multi-target analytical methods over wide concentration ranges is a real challenge. To date, the established method for this purpose is liquid chromatography coupled with tandem mass spectrometry (LC-MS/MS) [13,14,15,16]. However, the literature provides a limited number of methods describing a multi-target analysis of TKIs [17,18,19,20], mainly with a substantial sample pre-treatment through extraction before LC-MS/MS analysis and a significant sample volume. 

Multi-analyte assay methods help enhance laboratory workload efficiency by avoiding the need for multiple analytical methods, thereby reducing result turnaround time. The analytical development stage becomes a key challenge to cover a concentration range suitable for each compound. The same reasoning applies to simplifying sample pre-treatment (protein precipitation vs. extraction). Another limitation in TKI quantification in the pediatric population is the required sample volume for testing. Developing a method with a low sample volume requirement is particularly relevant in the case of TKI quantification in children.

Thus, we have developed and validated a rapid and sensitive analytical method for the simultaneous determination of 12 TKIs, including two metabolites, in LC-MS/MS: alectinib and its metabolite alectinib-M4, binimetinib, cobimetinib, crizotinib, dabrafenib, encorafenib, imatinib, lorlatinib, osimertinib and its metabolite AZ5104, and trametinib, with a sample intake of only 50 µL. The selection of these 12 drugs was based on the needs of clinicians. Their clinical indications and target concentrations are outlined in Table 1 [21,22,23,24,25,26,27,28,29,30].

This method was applied in both a pediatric population undergoing treatment for LCH with trametinib, dabrafenib, cobimetinib, or binimetinib, as well as in an adult population with cancer. 

The primary objectives were to determine target plasma concentrations within these groups and enhance our understanding of the effectiveness and safety profiles of these compounds. This is especially pertinent for children, as data concerning their usage remain extremely limited.

## 2. Materials and Methods

### 2.1. Materials and Reagents

The reference materials for alectinib, alectinib M4, AZ5104 (osimertinib active metabolite), crizotinib-13C2D5, imatinib, imatinib-D8, and osimertinib were purchased from LGC Standards (Molsheim, France). Crizotinib, lorlatinib, methanol, and ammonium formate were obtained from Sigma-Aldrich (St-Quentin-Fallavier, France). Alectinib-D8, binimetinib, binimetinib-13CD4, cobimetinib, cobimetinib-13C6, dabrafenib, dabrafenib-D9, encorafenib, encorafenib-13C2D3, osimertinib-13CD3, trametinib, and trametinib-13C6 were provided from Alsachim (Illkirch Graffenstaden, France). Acetonitrile and formic acid came from VWR. Ultra-pure water (18 MΩ) was produced via ultrafiltration with a Q-Pod (Millipore Corp., Molsheim, France). All chemicals were of analytical grade. Blank human plasma samples were provided by a local blood bank (EFS, Versailles, France) and stored at −20 °C until analysis. 

### 2.2. Calibration Standards and Quality Controls

Stock solutions at 1 g/L of the 12 TKIs (including two metabolites) and the 9 internal standards (IS) were prepared in DMSO/methanol (1:1, *v*/*v*). Subsequently, these solutions were further diluted in methanol to obtain working solutions at 125 mg/L for dabrafenib, encorafenib, and imatinib; 50 mg/L for alectinib, alectinib M4, binimetinib, cobimetinib, crizotinib, lorlatinib, and osimertinib; and 5 mg/L for AZ5104 and trametinib. Adapted volumes of these solutions or diluted solutions were spiked in blank plasma to create seven calibration standards, each featuring different concentrations according to the TKI under consideration. 

Isotope-labeled reference materials were prepared to match the concentrations of their corresponding non-labeled counterparts. In the absence of ready-to-use external quality controls (QC) specific to the investigated TKIs, a series of four levels of in-house QC were performed using separate stock solutions. Three concentration groups were established for calibration standards and the four levels of QC (Table 2). For each compound (except binimetinib and encorafenib), inter-laboratory QCs from a partner laboratory were analyzed to validate the internal QC levels. In addition, an External Quality Assessment (EQA) program was followed on a quarterly basis (Asqualab, Paris, France).

### 2.3. Sample Preparation

For each calibration sample, an adapted volume of working solution was added and evaporated. Then, 150 µL of IS methanolic solution was added to 50 µL of plasma sample (blank plasma for calibration sample or patient plasma) to precipitate proteins. After vortex mixing and 10 min of centrifugation, the supernatant was transferred into injection vials, and 10 µL was injected into the chromatographic system.

### 2.4. Instrumental Analysis

Chromatography was performed on a Dionex Ultimate 3000 Pump (ThermoFisher, les Ulis, France) using a Kinetex C18 Polar column (100 × 2.1 mm, 2.6 µm particle size) heated at 40 °C. The mobile phase A was made with 2 mM of ammonium formate in 0.1% formic acid in ultra-pure water and stored at +4 °C for a maximum of one week. The elution was achieved with a gradient of mobile phase A and acetonitrile (B) in 5 min at a flow rate of 400 µL/min. The gradient was set as follows: 10 to 40% of B from 0 to 1 min, then 40 to 90% of B in 1 min, and keeping this for 2 min before resetting to 10% B in 1 min. An equilibration time of one minute at 10% mobile phase B at the end of the chromatographic run allowed the column to be equilibrated before the next analysis.

The compounds were detected using an Altis^®^ triple quadrupole tandem mass spectrometer (ThermoFisher Scientific, Les Ulis, France) equipped with an electrospray ionization (ESI) source set in positive ionization mode with a potential of +3.5 kV. Nitrogen was used as cladding and auxiliary gas at a pressure of 30 and 10 arbitrary units (AU), respectively. The collision gas, argon, was used at a pressure of 1.5 mTorr. The temperature of the ion transfer capillary was set at 350 °C. Data were collected in multiple reaction monitoring mode (MRM) with two transitions (*m*/*z*) per compound. The MS parameters of the compounds are shown in Table 3. Xcalibur^®^ (v 4.2.47) and LC Quan^®^ (v 2.7.0) software (ThermoFisher Scientific) were used for data acquisition and processing.

### 2.5. Method Validation

#### 2.5.1. Limits of Detection and Quantification

The limit of detection (LOD) represents the lowest signal detected with a signal-to-noise ratio greater than 3. The limit of quantification (LOQ) was the lowest concentration measured with an accuracy of 80–120% and a coefficient of variation (CV) within ±20% [31,32].

#### 2.5.2. Linearity

Six calibration curves with seven calibration standards were analyzed on six different days.

The calibration curve was constructed by plotting the peak area ratio of each TKI to its corresponding internal standard (IS) against the nominal concentration of the corresponding calibration standard (CS).

Back-calculated concentrations of each calibration standard must fall within the range of 85–115% of their theoretical concentrations. However, an acceptable accuracy range of 20–80% was applied for the first calibration standard corresponding to the LOQ.

#### 2.5.3. Accuracy and Precision

Accuracy (bias %) and precision (coefficient of variation CV %) were evaluated for the four QC levels. Six replicates of each QC level were processed on the same day (intra-day assay) and on three different days (inter-day assay). Accuracy between 85 and 115% of the nominal concentrations and precision with a CV of less than 15% were required.

#### 2.5.4. Selectivity and Carryover

Drug-free blank plasma was analyzed to determine whether endogenous components may interfere with the retention times and the ion channels of TKI and IS. To evaluate carryover, a blank plasma sample was injected into LC-MS/MS immediately after the highest calibration standard. The area under the curve (AUC) ratio between this blank and LOQ must be less than 20% for TKIs and less than 5% for IS.

#### 2.5.5. Matrix Effect and Recovery

Matrix effect was evaluated using the ratio of the peak area obtained by analyzing 6 different blank plasma matrices spiked after precipitation with TKIs and IS at two concentrations (QC2 and QC4) to the peak area obtained in aqueous solution at the same concentrations. The internal standard normalized matrix effect (NME) was calculated using the following formula:

$$NME=\frac{matrix\;effect\;for\;analyte}{matrix\;effect\;for\;IS}\times 100\;(\%)$$

and was expressed as a “mean value” in the results, associated with the CV, which should not exceed 15%.

Recovery was evaluated using the ratio of the mean peak area obtained by analyzing 6 different blank plasma matrices spiked before precipitation with TKIs and IS at two levels (QC2 and QC4) to the mean peak area obtained in blank plasma spiked after precipitation at the same concentrations. 

The CV of the recovery calculated from the 6 lots of matrix should not be greater than 15%.

#### 2.5.6. Stability

To evaluate TKI stability in methanolic solutions, the four QC levels were realized with seven-day-old solutions and were analyzed six times. The accuracy and precision of the samples have to be in conformity with less than 15% variation.

TKIs stability in plasma was studied by analyzing the four QC levels 48 h after preparation (stored at +4 °C).

In addition, the stability of TKIs in plasma was assessed with an in-depth study of the literature and by analyzing samples from the quarterly external quality assessment program (Asqualab). These samples were received frozen at the beginning of the year and were analyzed after a period ranging from one to nine months after receipt, depending on the survey deadlines.

### 2.6. Application

#### 2.6.1. Population

This LC-MS/MS method has been routinely applied in our laboratory to children with histiocytosis and adults with melanoma or non-small cell lung cancer. The study was conducted in accordance with the Declaration of Helsinki and approved by the local ethics committee. Pediatric patients were included in their respective national LCH registries (Eudract 2015-000403-6), and informed consent was obtained from their parents for enrollment in this observational study. The current French registry has a CCTIRS notice e09 6 191, and the CNIL number is 909027. Adult patients were included after informed consent was obtained according to APHP (Assistance Publique des Hôpitaux de Paris, https://recherche.aphp.fr/eds/droit-opposition, accessed on 8 November 2023) data collection guidelines.

A total of 1021 residual plasma samples were analyzed. A total of 310 assays of dabrafenib, 422 assays of trametinib, 86 assays of cobimetinib, 100 assays of binimetinib, and 77 assays of encorafenib, as well as 3 assays of alectinib and its metabolite (alectinib M4), 13 assays of imatinib, and 10 assays of osimertinib (and its metabolite AZ5104), have been performed until June 2023. Populations treated with each TKI are described in Table 4.

#### 2.6.2. Sample Collection

Blood samples were collected in a steady state (i.e., a minimum of 2 weeks after the initiation of therapy or dose adjustment) just prior to dosing. The samples were collected in lithium heparin tubes without a separator and were immediately centrifuged, decanted, and frozen at −20 °C before LC-MS/MS analysis. 

#### 2.6.3. Statistical Analysis

Data were described by their central position (median and mean) and dispersion (interquartile range and confidence interval 95%) parameters. A Mann–Whitney *t* test was performed to compare dose–weight-adjusted concentrations between adult and pediatric groups. Results are illustrated in box-and-whiskers plots. GraphPad Prism v.8.3.0 Software (San Diego, CA, USA) was used to process and visualize data.

## 3. Results

### 3.1. Method Validation

The validation settings are presented in Table 5. The LOD was determined between 0.1 ng/mL for AZ5104 and 2.5 ng/mL for imatinib. The LOQ was between 0.2 ng/mL for AZ5104 and 10 ng/mL for crizotinib, with a bias of less than 19% and a CV of less than 17%.

The method exhibits good linearity using a linear regression model with a 1/x weighting factor. 

The intra-day accuracy ranges for the four QC samples were within the range of 85.1% to 113.7%, with a precision of less than 10.8% (CV). For inter-day analysis, the accuracy ranges were found to be between 87.5% and 109.8%, while the precision remained below 14.9% (CV).

**Figure 1:**
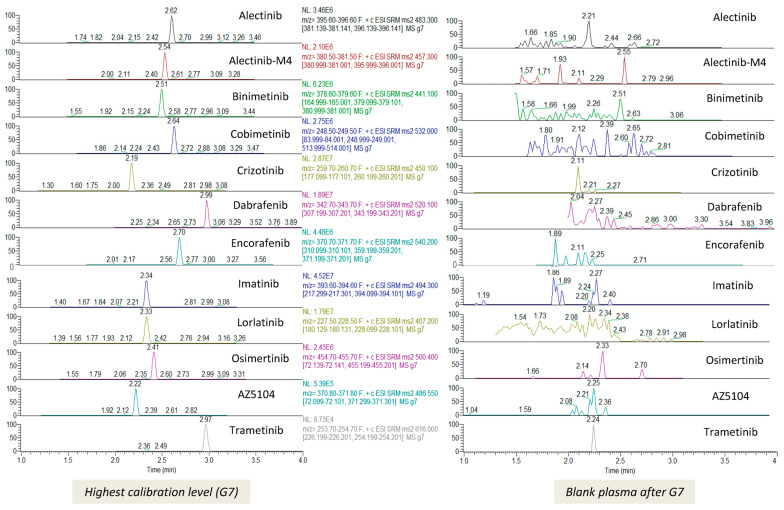
Chromatograms of the highest calibration level (G7) and of the blank sample just after G7 for the 12 TKIs. No carryover was observed. The area under the curve (AUC) ratio between the blank plasma after G7 and LOQ was less than 20%.

At the retention times of TKI and IS, no interfering signals were observed. Additionally, there was no carryover from the highest calibration standard. Chromatograms are presented in Figure 1. 

The matrix effect showed signal extinction for alectinib, binimetinib, cobimetinib, and imatinib and their IS. The IS normalized matrix effects, reported in Table 5, were compliant for all TKIs, with values between 70 and 130% (except for AZ5104, the osimertinib metabolite, for which the NME was 220% and 210% for CQ2 and CQ4, respectively). The IS normalized matrix effect CV was below 15% for all TKIs.

The recovery CV was also in agreement with the guidelines, except for the low QC (QC2) of crizotinib with a CV of 17.6%.

**Figure 2:**
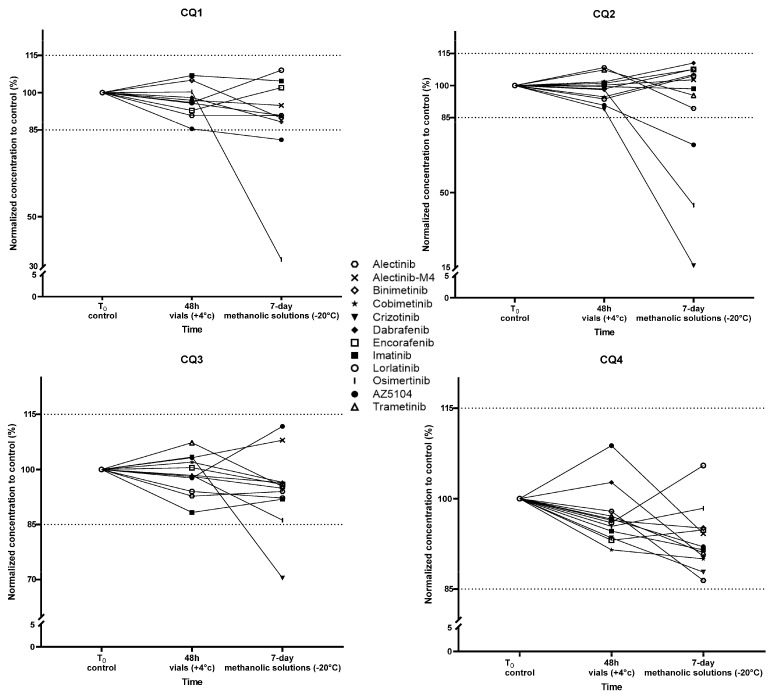
Data on stability for the 12 TKIs for 48 h in vials (ready-to-inject) at +4 °C and prepared with 7-day methanolic solutions at −20 °C for the 4 QC levels.

All TKIs were stable for 48 h in plasma at +4 °C. The mean bias (accuracy) of the four analyzed QCs was below 10.2% (imatinib). For the assessment of the stability of TKI methanolic solutions over a period of 7 days, the bias was below 15%, except for osimertinib and its metabolite AZ5104 and for crizotinib. Stability at −20 °C for 7 days improved with increasing concentrations. In fact, all the compounds were stable at −20 °C for 7 days for the highest CQ (CQ4) only, and crizotinib alone was not stable for CQ3. The data on stability are presented in Figure 2.

In addition, the laboratory participated in a quarterly external quality assessment program. All survey samples were received frozen at the beginning of the year and analyzed after a period of between one and nine months. The results were consistent. The last survey z-scores for each TKI are presented in Table 6.

### 3.2. TKIs Plasma Concentrations

TKIs plasma concentrations (median, mean, interquartile range, and confidence interval 95%) for adults and children are presented in Table 7. A total of 1021 assays were conducted, concerning 10 out of the 12 TKIs (dabrafenib, trametinib, cobimetinib, binimetinib, encorafenib, imatinib, alectinib and its metabolite alectinib M4, and osimertinib and its metabolite AZ5104).

**Figure 3:**
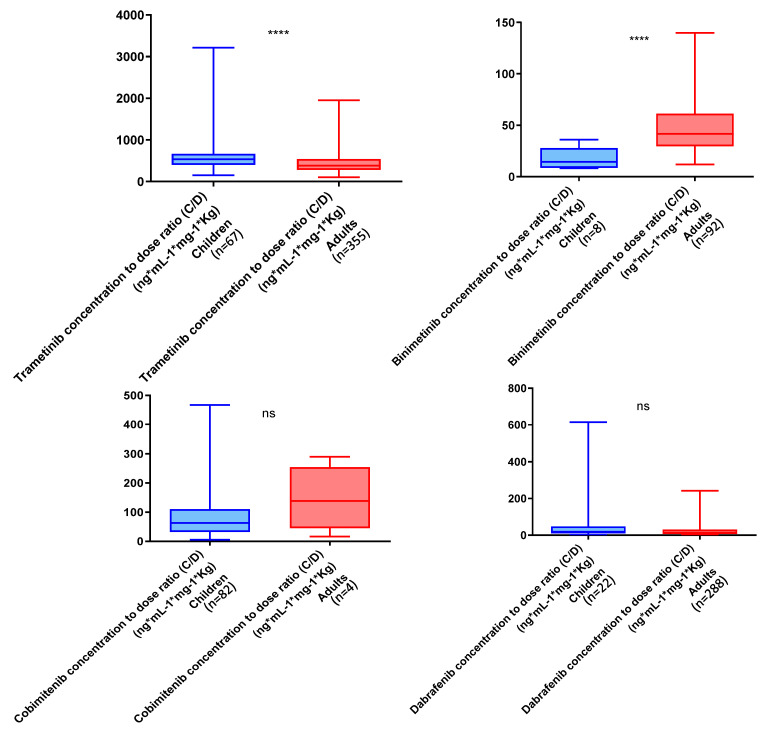
Comparison of trametinib, binimetinib, cobimetinib, and dabrafenib dose–weight-adjusted concentration (ng/mL/mg/kg) between pediatric and adult groups; (****): *p*-value < 0.0001 and ns: not statistically significant.

The dose–weight-adjusted concentrations of trametinib were significantly lower in the adult group than in the pediatric group, in contrast to the higher concentrations of binimetinib in adults (*p*-value < 0.0001). The dose–weight-adjusted concentrations of dabrafenib and cobimetinib were not statistically different between the two groups. The results of the statistical analyses comparing dose–weight-adjusted concentrations between the adult and pediatric groups are shown in Figure 3.

## 4. Discussion

The LC-MS/MS method for the quantification of the 12 described TKIs (including 2 metabolites) has been successfully validated in terms of linearity, LOD and LOQ, accuracy, precision, selectivity, carryover, matrix effect, recovery, and stability. 

Several analytical methods for assaying one or more TKIs have been reported in the literature [7,20,33,34]. Nevertheless, there is very little concern about 12 TKIs or more simultaneously. 

The method presented here was carried out on a very small sample volume of 50 µL, which is considerably less than previous publications analyzing more than 10 TKIs, such as the 300 µL used by Merienne et al. and Rousset et al. [19,35]. This is a major advantage, as sample quantity can be a limiting factor in analysis, especially in children. In addition, sample pre-treatment consisted of a simple methanol precipitation in the presence of the internal standard. The methods described in the literature are more elaborate, involving either liquid–liquid [17] or solid-phase [19] extractions. An additional advantage lies in the short analysis time, spanning just 5 min.

The concentration ranges were dynamically tailored to each compound, aligning with the therapeutic concentrations described in the literature [12,35]. 

The validation parameters were acceptable, with limits of quantification often lower than those found in the literature [19,34,36].

The matrix effect led to signal extinction or amplification for certain compounds. The matrix effect strictly depends on the interactions between the analyte and the interfering compounds in the matrix, based on the structure of the compounds. The matrix effect may also depend on the concentration of the analyte [37]. The relevance of the relative matrix effect (IS normalized matrix effect, or NME) has been extensively demonstrated by Nicolò et al. [38]. The evaluation of NME is the best indicator of the method’s ruggedness in terms of matrix effect for each analyte, with analogs IS or stable-isotope-labeled IS. Hence, in presenting our results, we opted to highlight the relative matrix effect (NME) rather than the absolute matrix effect for each TKI.

While variations in the matrix effect were observed for certain TKIs (especially crizotinib and lorlatinib) depending on the QC level, the IS normalized matrix effect CV of the 12 TKIs was below 15%, thus complying with the guidelines [31]. Furthermore, the use of stable-labeled isotopes (SLI) as internal standards effectively compensated for this extinction/amplification in the majority of TKIs, except for AZ5104, for which no SLI was accessible. Despite this, the normalized matrix effect (NME) was reproducible, and the developed method met established criteria for LLOQ, accuracy, and precision, enabling a reliable quantification of all 12 TKIs.

The precipitation recovery was assessed, with CV for each TKI under 15%, except for the low level of crizotinib CQ (17.6%). The variability observed for dabrafenib (CQ4) and encorafenib may be attributed to random error, which did not impact the performance of the validated method. Indeed, the accuracy and precision parameters, as well as the sensitivity of the four quality controls, were correct.

With regard to stability, the study was carried out according to the needs of the laboratory and the actual routine workload. It was shown that samples were stable for 48 h in vials (ready-to-inject) at +4 °C for all the TKIs analyzed. In addition, methanolic working solutions can be stored at −20 °C for 7 days, with the exception of osimertinib and its metabolite (AZ5104) and crizotinib, which must be prepared extemporaneously for each analysis.

Participation in a quality assessment program enabled us to demonstrate the stability of TKIs in plasma over several months at −20 °C, with z-scores < 2 for all the compounds analyzed (Table 6). Moreover, according to the literature data, trametinib serum stability was evaluated at 20 months at −20 °C and imatinib stability at one year at −20 °C [39,40]. The stability of crizotinib, alectinib, and osimertinib in plasma has been evaluated and validated for a minimum of one month [14,41]. Dabrafenib and cobimetinib are also described as stable in plasma at −40 °C for more than 20 weeks [42]. The frozen stability of binimetinib and encorafenib in plasma has been studied and found to be satisfactory for at least 30 days [43]. Lorlatinib seems to be stable in plasma for at least 2 months at −30 °C in plasma [44].

This method has been successfully applied to therapeutic drug monitoring (TDM) in both pediatric and adult populations undergoing treatment with TKIs. Its application has contributed to personalizing treatment for these patients and enhancing knowledge of optimal therapeutic concentration ranges for these drugs.

The TDM of TKIs is well established [12,13,45] and enables monitoring of both the efficacy and the safety of the treatment. Although the adverse effects of TKIs are mostly reversible and transient, they can sometimes lead to treatment interruption and a switch to another, better-tolerated treatment [10]. Digestive, cutaneous, and hematological disorders have been reported with imatinib [11]. Skin disorders (acneiform eruptions and rash) have been described for trametinib and dabrafenib [46]. Digestive disorders and hyperthermia may occur in patients treated with TKIs [25]. The determination of target concentrations is, therefore, of major interest in the management of patients treated with TKIs.

Furthermore, for the same dose, our study showed lower dose–weight-adjusted trametinib concentrations in adults than in the pediatric population. In contrast, binimetinib concentrations were higher in adults than in children (Figure 3). It should be noted, however, that the sample size in the two groups was different, which could introduce a bias. 

Age-related inter-individual variability has already been demonstrated for many treatments, but not for all TKIs [47,48]. Data are only available for vemurafenib [5,49]. Our study highlighted inter-individual variability between the adult and child groups for two TKIs (trametinib and binimetinib), which suggests the need for regular therapeutic drug monitoring for dose adjustment based on age.

Few data are available in the literature for children. Kondyli et al. reported signs of cutaneous toxicity, mainly associated with trametinib, in children treated for glioma, a finding supported by Selt et al. and Ronsley et al. [50,51,52]. No pharmacological data were reported in these studies. Furthermore, there are no available data regarding children with LCH, except for vemurafenib [5,53].

With regard to the data obtained in adults, presented in Table 1, there are no residual concentrations (Cmin) described in the literature for binimetinib, cobimetinib, and encorafenib. Therefore, the residual concentrations we have reported here bring new data for these compounds. The median concentrations of the remaining TKIs measured were in agreement with the concentrations reported in the literature (Table 1) and improved the pharmacokinetic data for these drugs in order to enhance the therapeutic management and follow-up of patients.

## 5. Conclusions

In conclusion, we have developed and validated a rapid and sensitive method for the simultaneous determination of 12 TKIs via LC-MS/MS (including two metabolites) with a sample intake of only 50 µL. This method was applied to pediatric and adult populations treated with TKIs. The validated method presented in this study proves its efficacy in analyzing clinically relevant concentrations over a wide range. Besides showcasing the significance of therapeutic drug monitoring for TKIs, especially in the pediatric population, the data gathered can aid in its meaningful interpretation. This method will, therefore, be used routinely in the laboratory in the management and monitoring of the efficacy and safety of TKIs in these sensitive populations. Consideration could be given to modifying the sample preparation to make it applicable to dried blood spots, a sampling method of particular interest for children, or when samples are collected in a geographical area distant from the analysis site, which can occur in our laboratory.

## Figures and Tables

**Table 1:** Characteristics of the 12 TKIs analyzed: tyrosine kinase target, indication in adults, and concentrations reported in the literature (NSCLC: non-small cell lung cancer).

Molecule	Target (TK)	Indication (Adults)	Target Concentrations (ng/mL)	Reference
Dabrafenib	BRAF	melanoma BRAF+ NSCLC BRAF+	Cmin ≥ 15	[21]
Trametinib	MEK	melanoma BRAF+ NSCLC BRAF+	Cmin ≥ 10.6	[22]
Binimetinib	MEK	melanoma BRAF+	Cmax = 654	[23]
Cobimetinib	MEK 1/2	melanoma BRAF+	Cmax = 270	[24]
Encorafenib	BRAF	melanoma BRAF+ metastatic colorectal cancer	Cmax = 3100	[25]
Osimertinib	EGFFR	NSCLC EGFR+	Cmin ≥ 90 (40 mg dosage) Cmin ≥ 160 (80 mg dosage)	[26]
AZ5104 (metabolite)	EGFR		-	
Crizotinib	ALK	NSCLC ALK+ ou ROS1+	Cmin ≥ 235	[27]
Alectinib	ALK, RET	NSCLC ALK+	Cmin ≥ 570	[28]
Alectinib M4 (metabolite)	ALK		Cmin ≥ 220	[28]
Imatinib	Bcr-Abl, c-Kit, PDGFR, SCFR, DDR	Chronic myeloid leukemia Acute lymphocytic leukemia Gastrointestinal stromal tumor Myelodysplastic/myeloproliferative syndromes	Cmin ≥ 1100	[29]
Lorlatinib	ALK ROS1	NSCLC ALK+	Cmax = 575	[30]

**Table 2:** Concentrations of calibration standards and quality controls (QC) for the three groups of TKIs (ng/mL).

Molecules	G1	G2	G3	G4	G5	G6	G7	QC1	QC2	QC3	QC4
AZ5104 (Osimertinib metabolite)	0.2	1	2	10	20	100	200	0.3	3	15	150
Trametinib											
Alectinib	2	10	20	100	200	1000	2000	3	30	150	1500
Alectinib M4 (metabolite)											
Binimetinib											
Cobimetinib											
Crizotinib											
Lorlatinib											
Osimertinib											
Dabrafenib	5	25	50	250	500	2500	5000	7.5	75	375	3750
Imatinib											
Encorafenib											

**Table 3:** Retention time (Rt), parent and fragment ions, and collision energies for the studied TKIs and IS.

Compound	Rt (min)	Parent Ion *m*/*z*	Fragment Ions *m*/*z*	Collision Energy (eV)
Alectinib	2.7	483.3	381.1	36
			396.1	23
Alectinib-D8	2.7	491.7	396.2	24
			381.1	36
Alectinib M4	2.6	457.3	381	33
			396	30
AZ5104	2.1	486.5	72.1	28
			371.3	32
Binimetinib	2.6	441.1	379.1	19
			165	33
Bimetinib-13CD4	2.6	449.1	381	19
			165	31
Cobimetinib	2.8	532	84	29
			249	30
Cobimetinib-13C6	2.8	538.1	84	29
			255	32
Crizotinib	2.2	450.1	177.1	38
			260.2	23
Crizotinib-13C2D5	2.2	457.2	177.1	42
			267.2	26
Dabrafenib	3.2	520.1	307.2	33
			343.2	26
Dabrafenib-D9	3.2	529.1	316.3	34
			352.3	28
Encorafenib	2.8	540.2	310.1	38
			371.2	36
Encorafenib-13C2D3	2.8	544.2	310	34
			371	37
Imatinib	2.3	494.3	217.3	25
			394.1	26
Imatinib-D8	2.3	502.4	225.1	26
			394.1	27
Lorlatinib	2.3	407.2	228.1	2
			180.1	23
Osimertinib	2.4	500.4	72.14	25
			455.2	21
Osimertinib-13CD3	2.4	504.7	389.2	34
			459.2	21
Trametinib	3.1	616	226.2	46
			254.2	37
Trametinib-13C6	3.1	622	232.2	48
			260.2	39

**Table 4:** Characteristics of TKI-treated patients monitored in our laboratory from 2014 up to June 2023.

	Dabrafenib		Trametinib		Cobimetinib		Binimetinib		Encorafenib	Alectinib	Imatinib	Osimertinib
	Adults	Children	Adults	Children	Adults	Children	Adults	Children	Adults	Adults	Adults	Adults
Number of patients	95	8	106	10	4	14	24	1	20	1	7	9
Average age (min–max) (years)	58 [25–90]	11 [3–16]	62 [43–90]	9 [1–17]	55 [23–86]	3.2 [0.1–13]	62 [33–84]	2	60 [33–83]	66	60 [40–81]	75.5 [45–86]
Sex ratio	1.2	1	2.3	2.3	3	1	1.6	-	0.6	-	1.3	0.3
Average dose (min–max) (mg/kg)	4 [1.5–7.3]	2 [0.4–4.7]	0.028 [0.01–0.05]	0.023 [0.01–0.05]	0.5 [0.3–0.6]	1.2 [0.9–2.7]	1.2 [0.8–2.4]	6.9	5.8 [3.8–12.2]	21	4.5 [1.4–7.5]	1.2 [0.6–2]
Number of analyzed samples	288	22	355	67	4	82	77	8	77	3	13	10

**Table 5:** Validation parameters of the 12 TKIs. Normalized matrix effect CV and recovery CV were calculated for the 6 lots of plasma. LOD = limit of detection, LOQ = limit of quantification, and CV = coefficient of variation.

Molecules	LOD (ng/mL) (*n* = 6)	LOQ (ng/mL) (*n* = 6)	Linearity Range (ng/mL) (*n* = 6)	R^2^	Accuracy (Bias, %)		Precision CV (%)		Recovery (*n* = 6)				Normalized Matrix Effect (NME) (*n* = 6)			
					Intra-Day (*n* = 6)	Inter-Day (*n* = 18)	Intra-Day (*n* = 6)	Inter-Day (*n* = 18)	QC2		QC4		QC2		QC4	
									Mean Value	CV (%)	Mean Value	CV (%)	Mean Value	CV (%)	Mean Value	CV (%)
Dabrafenib	0.5	5	5–5000	0.9984	88.3–103.7	89–102.9	3.5–5.5	2.1–8.4	103.5	14.2	70.1	9.1	100	9.4	100	5.1
Trametinib	0.5	1	1–200	0.9980	91.8–111.3	92.4–106.3	4.7–10.8	2–14.9	94.8	10.4	85.1	6.4	120	7.9	100	6.3
Cobimetinib	0.4	2	2–2000	0.9986	88.3–103.1	89.6–102	4.7–7.3	2.5–4.9	97.8	5.8	113.8	7.1	120	3.2	100	5.5
Encorafenib	1	5	5–5000	0.9990	90.8–106.3	92.8–103.2	3.9–7.4	5.2–14.8	88.3	15.1	114.9	8.8	110	10.8	100	9.9
Binimetinib	0.2	2	2–2000	0.9989	89.5–103.1	92.7–99.5	3.4–6.7	2.5–8.4	110	11.1	107.7	10	100	8.6	100	6.5
Osimertinib	2	2	2–2000	0.9959	96.9–113.7	100.3–109.8	4.3–8.1	5.6–7.9	106.1	10	98.3	12.5	100	7.5	120	12.8
AZ5104	0.1	0.2	0.2–200	0.9987	88.7–113.6	93.8–107.4	5.8–9.8	9.9–14.6	109.8	9	112.5	11.8	220	8.9	210	8.8
Crizotinib	2	10	10–2000	0.9974	85.1–109	87.5–108.5	2.8–6.3	1–12.3	119.7	17.6	118.0	10.5	130	8.6	100	5.5
Alectinib	0.2	2	2–2000	0.9984	85.4–103.9	89.8–102.4	5.3–8.8	1.9–14.4	106.4	12.1	113.1	13.8	70	6.7	70	10.9
Alectinib M4	0.2	2	2–2000	1	86.8–106.3	91.3–103.1	6.4–10.2	11.2–11.8	107.8	4	113.1	9.8	110	4.8	110	9.1
Imatinib	2.5	5	5–5000	0.9990	88.7–97.5	92–94.9	3.9–5.9	6.1–11	109.3	6	111.6	9.2	120	3.3	110	6.7
Lorlatinib	0.2	2	2–2000	0.9980	89.5–105.3	95.7–99	3.7–6.9	8–14.6	104.6	8.1	114.2	14.5	80	5.3	110	15.1

**Table 6:** Results (z-score) of Asqualab external quality assessment program (ITK2301). Acceptable Z-score < 2.

Compound	Measured Value (µg/L)	Mean (µg/L)	Z-Score
Alectinib	296	316	−0.27
Binimetinib	150	137	0.64
Cobimetinib	74	64.6	1.12
Crizotinib	78	72.8	1.3
Dabrafenib	58	53.1	1.1
Encorafenib	50	44.1	0.94
Imatinib	528	446	1.38
Trametinib	5.5	4.74	0.98

**Table 7:** Descriptive statistics of 10 TKIs for adults and children: median concentrations, interquartile range (IQR), and mean and confidence interval (CI) 95% in ng/mL.

	Dabrafenib		Trametinib		Cobimetinib		Binimetinib		Encorafenib	Alectinib	Alectinib M4	Imatinib	Osimertinib	AZ5104
	Adults (*n* = 288)	Children (*n* = 22)	Adults (*n* = 355)	Children (*n* = 67)	Adults (*n* = 4)	Children (*n* = 82)	Adults (*n* = 92)	Children (*n* = 8)	Adults (*n* = 77)	Adults (*n* = 3)	Adults (*n* = 3)	Adults (*n* = 13)	Adults (*n* = 10)	Adults (*n* = 10)
Concentration range CI 95% (ng/mL)	[88–122]	[26–178]	[11–13]	[11–14]	[24–94]	[74–118]	[51–64]	[37–204]	[16–21]	[292–424]	[125–170]	[1132–1702]	[122–320]	[16–56]
IQR (ng/mL)	[29–114]	[15–131]	[8–14]	[6.5–17]	[46.5–83]	[34–114]	[38–69]	[62–143]	[10–22]	[302–408]	[132–168]	[1254–1742]	[95–321]	[11–47]
Median concentration (ng/mL)	53.5	18.4	11	9.7	70	64.3	52	100	15	346	156	1618	207	25
Mean concentration (ng/mL)	105.3	102.3	12.4	12.6	59.3	96.3	57.4	120	18.6	358	147	1417	221	32
Dose–weight-adjusted median concentration (ng/mL/mg/kg)	14	19.1	386.3	538.5	138.1	62.6	41.6	14.6	2.6	16.7	7.5	321.7	211.4	21.7
Dose–weight-adjusted mean concentration (ng/mL/mg/kg)	28.3	62.3	443.8	578.4	145.7	85.4	48.8	17.5	3.4	17.3	7.1	363.6	196.7	25.8

## Data Availability

Data will be available on request.
